# Analytical Performance Specifications for 25-Hydroxyvitamin D Examinations

**DOI:** 10.3390/nu13020431

**Published:** 2021-01-28

**Authors:** Etienne Cavalier, Callum G. Fraser, Harjit P. Bhattoa, Annemieke C. Heijboer, Konstantinos Makris, Candice Z. Ulmer, Hubert W. Vesper, Samuel Vasikaran, Pierre Lukas, Pierre Delanaye, Anna Carobene, on behalf of the IFCC-IOF Committee for Bone Metabolism

**Affiliations:** 1 CHU de Liège and Centre de Recherche Intégré sur les Médicaments (CIRM), Department of Clinical Chemistry, University of Liège, Domaine du Sart-Tilman, B-4000 Liège, Belgium; pierre.lukas@chuliege.be; 2 Centre for Research into Cancer Prevention and Screening, Ninewells Hospital and Medical School, University of Dundee, Dundee DD1 9SY, UK; c.g.fraser@dundee.ac.uk; 3 Department of Laboratory Medicine, Faculty of Medicine, University of Debrecen, H-4032 Debrecen, Hungary; harjit@med.unideb.hu; 4 Endocrine Laboratory, Department of Clinical Chemistry, Amsterdam Gastroenterology & Metabolism, Vrije Universiteit Amsterdam and University of Amsterdam, Amsterdam UMC, 1081 HVAmsterdam, The Netherlands; a.heijboer@amsterdamumc.nl; 5 Clinical Biochemistry Department, KAT General Hospital, GR-14561 Athens, Greece; kostas.makris.km@gmail.com; 6 National Center for Environmental Health, Centers for Disease Control and Prevention, Division of Laboratory Sciences, Atlanta, GA 30329, USA; obz0@cdc.gov (C.Z.U.); hav2@cdc.gov (H.W.V.); 7 PathWest Laboratory Medicine, Fiona Stanley Hospital, Murdoch 6150, Australia; Samuel.Vasikaran@health.wa.gov.au; 8 Department of Nephrology, Dialysis and Transplantation, University of Liege, CHU de Liege, B-4000 Liège, Belgium; pierre_delanaye@yahoo.fr; 9 Department of Nephrology-Dialysis-Apheresis, Hôpital Universitaire Caremeau, 30900 Nîmes, France; 10 Laboratory Medicine, IRCCS San Raffaele Scientific Institute, 20132 Milan, Italy; carobene.anna@hsr.it

**Keywords:** analytical performance specifications biological variation, measurement uncertainty, vitamin D, 25(OH)-vitamin D

## Abstract

Currently the 25-hydroxy vitamin D (25(OH)D) concentration is thought to be the best estimate of the vitamin D status of an individual. Unfortunately, its measurement remains complex, despite recent technological advances. We evaluated the biological variation (BV) of 25(OH)D in order to set analytical performance specifications (APS) for measurement uncertainty (MU). Six European laboratories recruited 91 healthy participants. The 25(OH)D concentrations in K_3_-EDTA plasma were examined weekly for up to 10 weeks in duplicate on a Lumipulse G1200 (Fujirebio, Tokyo, Japan). The linear regression of the mean 25(OH)D concentrations at each blood collection showed that participants were not in a steady state. The dissection of the 10-sample collection into two subsets, namely collections 1–5 and 6–10, did not allow for correction of the lack of homogeneity: estimates of the within-subject BV ranged from 5.8% to 7.1% and the between-subject BV ranged from 25.0% to 39.2%. Methods that would differentiate a difference induced by 25(OH)D supplementation at *p* < 0.05 should have MU < 13.6%, while at *p* < 0.01, the MU should be <9.6%. The development of APS using BV assumes a steady state of patients. The findings in this study suggest that patients are not in steady state. Therefore, APS that are based on MU appear to be more appropriate.

## 1. Introduction

Twenty-five-hydroxy vitamin D (25(OH)D) measurement remains complicated despite recent technological advances [1]. Indeed, 25(OH)D assays must recognize both 25(OH)D2 and 25(OH)D3 equivalently; 25(OH)D is very lipophilic and needs to be removed from its major carrier form, vitamin D binding protein (DBP), and physiological or pathological conditions such as pregnancy, estrogen therapy, or renal failure often lead automated immunoassays to fail in correctly quantitating 25(OH)D [2]. On the other hand, the standardization of 25(OH)D measurements is an ongoing process led by the International Federation of Clinical Chemistry and Laboratory Medicine (IFCC), the Vitamin D Standardization Program (VDSP), and the Centers for Disease Control (CDC) Vitamin D Standardization Certification Program (VDSCP) [3]. This process is a prerequisite for the achievement of comparable results across different measurement methods and across manufacturers.

The aim of these standardization programs is that 25(OH)D measurements are accurate, as well as comparable over time, location, and laboratory procedure, to the concentrations obtained using Joint Committee for Traceability in Laboratory Medicine (JCTLM) recognized reference measurement procedures (RMPs), such as those operated at the National Institute of Standards and Technology (NIST) [4], Ghent University [5], or the CDC [6]. By linking the measurements performed in patient care to these RMPs, metrological traceability as outlined in the International Organization for Standardization (ISO) document 17511 can be achieved. ISO 17511 does not provide analytical performance specifications. These have been proposed by the clinical laboratory community and have been adopted in these standardization programs. Assays are considered traceable to International System (SI) units when they are calibrated to a JCTLM—recognized RMP, and they are considered standardized when their calibration bias, expressed as mean bias, is <5% to the RMP and the assay imprecision, expressed as mean coefficient of variation, is <10% [7]. The CDC Vitamin D Standardization-Certification Program (VDSCP) determines the calibration bias and imprecision by evaluating the data obtained from 40 blinded single-unit, fresh-frozen serum samples measured in batches of 10 samples over four consecutive quarters. With improvements in calibration bias among assays, the measurement bias observed in individual samples became more apparent. To assist assay manufacturers and users of assays, the CDC VDSCP started to provide information on individual sample pass rates, which represents the proportion of the 40 samples that met the bias criterion (<5%). In June 2020, 34 methods, from either in-vitro diagnostics (IVD) manufacturers or in-house methods developed in medical laboratories, were considered certified by CDC VDSCP and standardized against the RMP for the year 2019. However, the “individual sample pass rate” in 2019 was quite different from one method to the other and ranged from 45% to 88% with liquid chromatography tandem mass spectrometry (LC-MS/MS) methods (mean pass rate of 63%) and from 8% to 68% for immunoassays (mean pass rate of 30%). The list of these standardized participants and their respective methods can be found on the VDSCP portion of the CDC’s external website [8].

The criteria for an acceptable method of CV < 10% and a mean bias < 5% were taken from Stöckl et al. [7], who used data from multiple studies and diverse study populations. With the aim of defining analytical performance specifications (APS) for a reference measurement system for 25(OH)D, four of the five models in the hierarchy set at the 1999 Stockholm Consensus Conference on Setting Global Analytical Goals in Laboratory Medicine [9] were reviewed. The models were as follows: (1)—misclassifications in diagnosis; (2)—biological variation (BV) data mainly derived from the reference interval (RI) but also from an evaluation of 25(OH)D monitoring: (3)—expert recommendations; and (4)—APS set by (a) regulatory bodies, (b) organizers of External Quality Assessment Schemes (EQAS), and (c) the state-of-the-art performance. The APS used in the VDSP and VDSCP are based on Model 2.

However, as the recommendations of Stöckl et al. were published in 2009 [7], there have been many developments that impinge on their conclusions. Firstly, there is a new simpler approach advocated by the European Federation of Clinical Chemistry and Laboratory Medicine (EFLM) to set APS using three models, namely: (1)—clinical outcomes, (2)—BV, and (3)—state-of-the-art performance [10]. Secondly, as shown in the EFLM BV database [11], there have been additional publications on the BV of 25(OH)D, but the results for the within-subject BV (CV_I_) range from 6.9% [12] to 21.2% [13], with the between-subject BV (CV_G_) being greater than 40% [14]. Thirdly, there has been significant improvement in the “state-of-the-art” performance over the last decade, mainly because of the various standardization activities. As a result, the application of Model 2, where the APS is based on BV, is favored [15]. This is also the case for the generation of APS for standard measurement uncertainty (MU) [16]. MU is a non-negative parameter characterizing the dispersion of the quantity values being attributed to a measurand, based on the information used. The measurement uncertainty typically accounts for the combined effect of random (i.e., imprecision) effects and remaining systematic (i.e., bias) uncertainty following imperfect correction. The aim of this study was to evaluate the BV of 25(OH)D in samples obtained in the large European Biological Variation (EuBIVAS) study [17], which recently adopted a recommendation for the generation of BV components [18], and to see if we could significantly update the model proposed by Stöckl et al. [7].

## 2. Materials and Methods

### 2.1. Biological Variation Data Derived from the EuBIVAS

The EuBIVAS has been described in detail elsewhere [17]. Briefly, six European laboratories located in Milan (Italy, 45.47° N, 9.19° E), Padua (Italy, 45.41° N, 11.87° E), Bergen (Norway, 60.39° N, 5.33° E), Madrid (Spain, 40.42° N, 3.70° W), Assen (The Netherlands, 52.99° N, 6.6° E), and Istanbul (Turkey, 41.01° N, 28.97° E) were involved. At the beginning of the study, 105 subjects were recruited. Three subjects were not included in the final cohort after the application of the inclusion/exclusion criteria during the first collection, and five people withdrew during the study for personal reasons.

**Figure 1:**
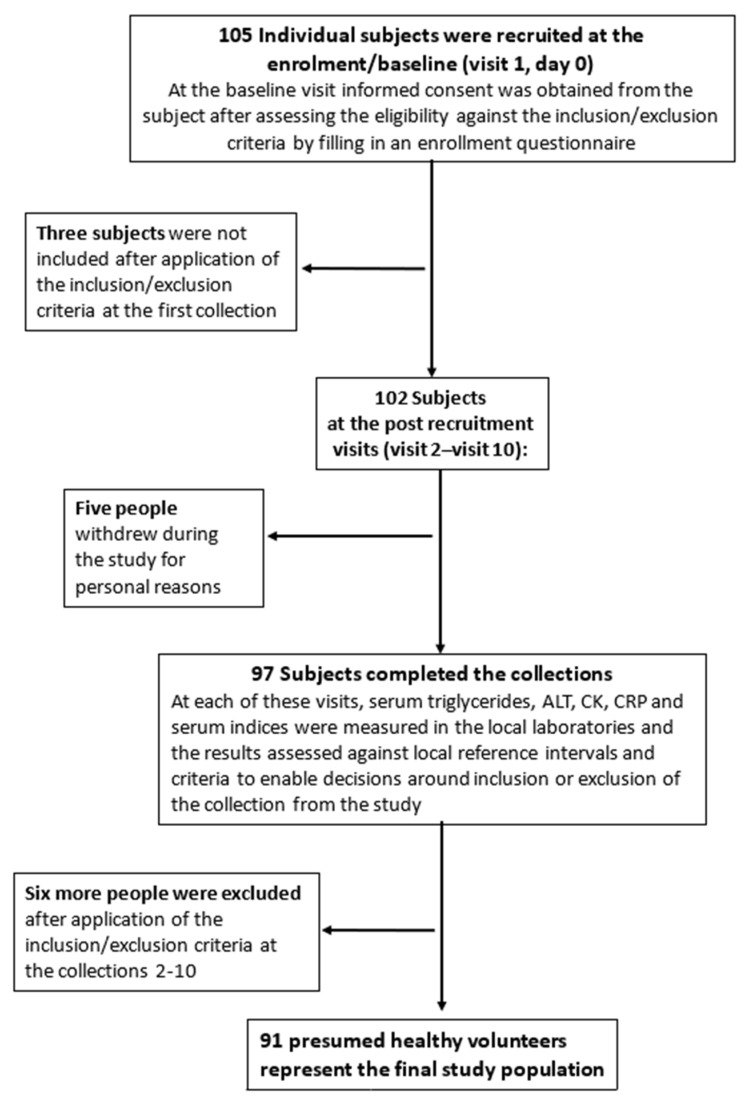
Selection of the participants for the European Federation of Clinical Chemistry and Laboratory Medicine European Biological Variation Study (EuBIVAS) study. ALAT: alanine aminotransferase; CK: creatinine kinase; CRP: C-reactive protein.

The final study population for sample collection consisted of 97 presumed healthy volunteers. Further exclusions from the final cohort were based on the laboratory measurements made at each visit. In particular, two males were excluded for suspicions of subclinical viral infection as a result of a significant negative trend in γ-glutamyl transferase (GGT) and alanine aminotransferase (ALT) activities; two further males were excluded because of raised creatine kinase (CK) and raised ALT on a number of occasions; another male for an unknown liver problem with elevated ALT during several collections; and a further male with elevated ALT (three collections), CK (one unusual value), and C-reactive protein (CRP) (three unusual concentrations) [17]. This led to further exclusion of six subjects, resulting in the recruitment of 91 presumed healthy participants (38 males and 53 females; age range of 21–69 years; Figure 1).

The participants completed an enrollment questionnaire to provide information on their lifestyle and presumed health status, which was further verified by a set of routine laboratory tests performed during each collection. One potential participant was taking vitamin D supplementation and was thus excluded. All laboratories followed the same protocol for the pre-examination phase. Fasting blood samples were drawn by venipuncture weekly for 10 consecutive weeks (April–June 2015) on a set day (Tuesday to Friday), and at the same time (e.g., between 08:00 a.m. and 10:00 p.m. at each weekly visit) by the same phlebotomist at most visits, further minimizing variation. The number of collections per subject means that the resultant data will be from studies of a high statistical power, delivering high quality estimates of the components of examination and biological variation with tight confidence intervals [19]. In total, 77 participants completed all 10 collections, 10 participants completed 9 collections, 2 participants completed 8 collections, and 2 participants completed 7 collections. The K_3_-EDTA plasma samples collected by each laboratory were sent frozen on dry ice to San Raffaele Hospital, Milan, Italy. The samples were stored in a −80 °C freezer until they were shipped on dry ice to the Centre Hospitalier Universitaire (CHU) de Liège, Belgium, where the 25(OH)D concentrations were determined in May 2020. Although we have no data on the five—year stability of 25(OH)D at −80 °C under our conditions, different pre-examination studies have shown that this measurand is remarkably stable, even under extreme conditions [20,21].

The study was approved by the Institutional Ethical Review board of San Raffaele Hospital (Milan, Italy; protocol number: WG-BV project #001, 50/INT 2014) in agreement with the World Medical Association Declaration of Helsinki (as revised in 2013) and by the Ethical board/ regional Ethics Committee for each involved center (protocol number: WG-BV project #001, PI-1993. April 2015 for Spain; WG-BV project #001, 2014-26 for The Netherlands; WG-BV project #001, 3452/AO/15 for PD Italy; 2015-3/17 for Turkey; 2014/1988 for Norway).

### 2.2. Data Analysis

The data analysis was performed as previously described [22,23]. Briefly, CV_I_ was estimated using one way analysis of variance (CV-ANOVA) for all participants, as well as for males and females separately. Outlier identification and removal were performed for replicates and samples on the CV-transformed data, by assessing homogeneity of the measurement CV (CV_A_; between-replicates) using the Bartlett test, and the homogeneity of CV_I_ using the Cochran test, as recommended and applied in the European Federation of Clinical Chemistry and Laboratory Medicine European Biological Variation Study (EuBIVAS) strategies [17]. The CV_G_ estimates were estimated on natural log-transformed data.

To examine if there was a general trend in the overall concentration over the study period, and if individual participants were in steady state, we calculated the regression of the mean of the 180 duplicate measurements from every blood draw (1, 2 …. 10) (pooled mean group sample concentrations) versus the blood draw number (1–10). Subjects were considered in steady state if the 95% confidence intervals (CI) of the slope of the regression line included zero.

BV data estimation after outlier exclusion and trend analysis were both performed for the whole data set derived from 10 blood collections, as well as for the data derived from the first five collections (April–May) and the data from the last five collections (May–June).

The results for the 25(OH)D mean values, CV_I_ and CV_G_, and the estimates between the male and female subgroups were considered significantly different if the associated 95% CI did not overlap. Data analyses were performed using Microsoft Excel 2010 (Microsoft, Redmond, WA, USA) and IBM SPSS (Chicago, IL, USA) v23.

### 2.3. Analytical Methods

We measured the 25(OH)D concentrations with the Fujirebio assay on a Lumipulse G1200 instrument (Fujirebio, Tokyo, Japan). This method is the only non-competitive immunoassay (sandwich) method for 25(OH)D measurement [24], the performance characteristics of which surpass the VDSP criteria for acceptability [7] and had an individual sample pass rate of 68% in 2019, according to the CDC VDSCP certification report [8]. All of the measurements were performed according to the manufacturer’s instructions in the clinical chemistry laboratory of CHU de Liège. The manufacturer’s internal quality control materials (two concentrations) were measured at the beginning and end of each run, following the validation of the calibration curves by the instrument. Samples donated from each participant were measured in duplicate within the same run on a single day.

## 3. Results

### 3.1. APS for 25(OH)D Based upon Biological Variation Derived from the EuBIVAS, EFLM Model 2

The median number of participants per center was 15 (range: 12–19). The participants were generally physically active and approximately 3% were regular smokers (the detailed demographic characteristics are documented in previous publications) [17,25]. Their median body mass index (BMI) was 22.5 kg/m^2^ (range of 17.6–32.5 kg/m^2^) and none of the participants suffered from renal impairment.

The overall mean 25(OH)D concentration was 19.0 (95% confidence interval (CI): 18.6–19.4%) ng/mL and the mean CV_A_, determined from the duplicate examinations, was 1.7% (95% CI: 1.6–1.8%). The differences in concentrations between males, with lower concentrations, and females were identified by the lack of overlap of 95% CIs, so that different CV_G_ were estimated for males and females, respectively. No significant differences between CV_I_ estimates between males and females were observed (Table 1).

**Figure 2:**
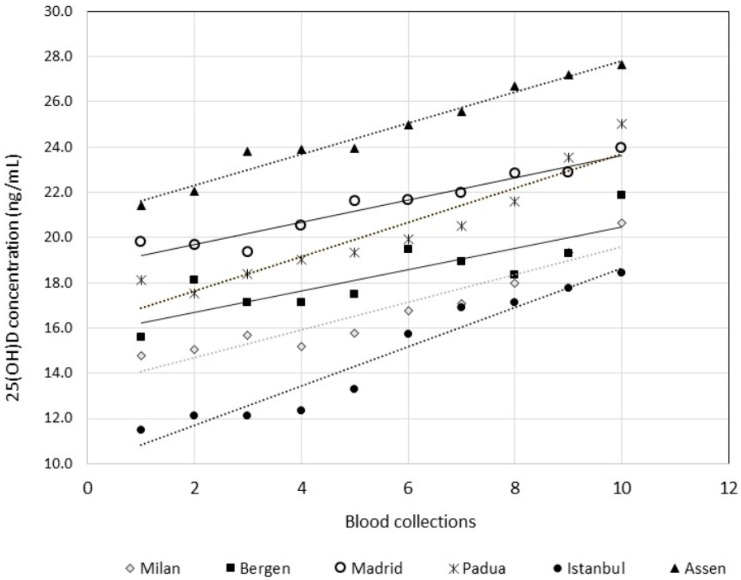
An x/y plot for vitamin D mean values (Y) versus blood collections (X), according to the countries. Linear regression equation and Pearson correlation coefficient (*r*) produced the following: Milan, Italy: Y (ng/mL) = 0.61 × (0.44 − 0.79) + 13.5 (12.4 − 14.5), *r* = 0.943; Bergen, Norway: Y (ng/mL) = 0.47 × (0.22 − 0.72) + 15.7 (14.2 − 17.3), *r* = 0.840; Madrid, Spain: Y (ng/mL) = 0.50 × (0.38 − 0.61) + 18.7 (18.0 − 19.4), *r* = 0.964; Padua, Italy: Y (ng/mL) = 0.76 × (0.54 − 0.98) + 16.1 (14.8 − 17.5), *r* = 0.944; Istanbul, Turkey: Y (ng/mL) = 0.87 × (0.68 − 1.06) + 9.9 (8.8 − 11.1), *r* = 0.967; Assen, The Netherland: Y (ng/mL) = 0.68 × (0.59 − 0.78) + 21.0 (20.4 − 21.5), *r* = 0.986.

A linear regression of the mean of all of the concentrations from each blood drawing against the blood drawing number was performed. This regression showed that the participants were not in a steady state with regard to 25(OH)D concentrations, as the 95% CI of the slope did not include zero. Figure 2 shows that, irrespective of the geographical location of the participants, 25(OH)D concentrations tended to linearly increase according to the time of sampling during this European Spring in 2015. Hence, this set of data could not be considered homogenous according to the Cochran test [26], a prerequisite for the estimation of CV_I_ and CV_G_. The homogeneity of the data was achieved only by eliminating more than 50% of the data, thus reducing the CV_I_ from 17.8% (whole population, original data) to 6.3% (95% CI: 5.9–6.8%; 41 subjects; Table 1). The dissection of the entire 10 sample collections into two subsets, i.e., the first set being collections 1 to 5, and the second set being collections 6 to 10, did not correct the lack of homogeneity issue, resulting in the detection of outliers in about 13% of the data. The CV_I_ ranged from 5.8% to 7.1% according to the sex and subset of the collection (Table 1). The CV_G_ ranged from 25.0% to 39.2%. These results clearly showed that the traditional BV concept, in which a measurand varied around a homeostatic setting point (CV_I_) with differences among homeostatic setting points (CV_G_), was quite inappropriate for 25(OH)D.

### 3.2. APS for 25(OH)D Based on the Effect of MU on Change in Serial Results, EFLM Model 1

**Figure 3:**
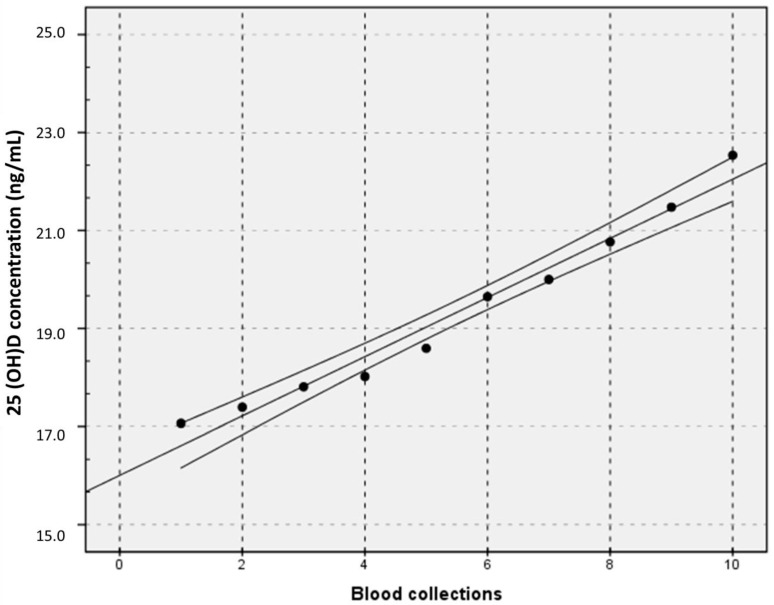
An x/y plot for vitamin D mean values (Y) versus blood collections (X). Linear regression equation with 95% CI: Y (ng/mL) = 0.604 × (0.52 − 0.70) + 16.0 (15.5 − 16.5); Pearson correlation coefficient (*r*) = 0.985.

Figure 2 shows that, although the mean 25(OH)D concentration for the group varied from geographical location to location (i.e., the intercepts differed), and although the 25(OH)D concentrations increased for every location at slightly different rates (i.e., the slopes differed), the data represent the “physiological” variation in these European participants during Spring 2015. Hence, the entire data set might be used for further assessment using a strategy that could be judged as an approach to EFLM Model 1 for the derivation of APS, a strategy based on the assessment of the effect of the examination performance on clinical outcomes. Figure 3 shows the regression of the mean of the 180 values of sample collection versus the study week. The mean difference in concentration between consecutive samples was 2.8%. An examination method that would be able to significantly differentiate a physiological weekly variation of 25(OH)D concentration should possess performance characteristics, i.e., measurement uncertainty (MU), that can be calculated as follows:

Change (%) = 2^1/2^ × MU (%) × Z

where Z is the Z-score, i.e., the number of standard deviations (SD) appropriate to the probability. In this case, the variation is an increase, so the statistical approach must be one-sided and for a 95% probability, Z is 1.645. So, 2.8 = 2^1/2^ × MU (%) × 1.645, and the APS of a method used to detect a significant physiological change, *p* < 0.05, should have a MU < 1.2%.

The attainment of this APS would be important for the use of an RMP. However, from a clinical perspective, there is no need to be able to meaningfully detect a week-to-week variation. Indeed, the interval between starting vitamin D supplementation and measuring/monitoring the 25(OH)D concentration should at least be three months [27]. The regression shows that the physiological variation of the 25(OH)D concentration in participants over a 10-week period was 31.6%. According to the equations provided above, that is, 31.6% = 2^1/2^ × MU (%) × 1.645, a method that would differentiate a physiological change from a change induced by vitamin D supplementation at *p* < 0.05, should have a MU < 13.6%. If it was deemed clinically necessary to be more certain that an increase had occurred, for example, at *p* < 0.01, the appropriate Z-score should be 2.326 so that the APS for the MU would be 9.6%, as the formula would become 31.6% = 2^1/2^ × MU (%) × 2.326.

## 4. Discussion

Vitamin D deficiency has become a worldwide problem [28]. The accurate determination of 25(OH)D to assess deficiency and its medication is thus mandatory, and VDSP and CDC VDSCP have been efficiently working over recent years to promote the standardization of the examination methods. It should be realized that all of these standardization efforts applied the APS developed by Stöckl et al. [7] and recommended by the clinical laboratory community. The strategies that were used to derive APS for RMP and for clinical diagnosis and monitoring were based on the internationally well-accepted hierarchy of approaches, which were agreed upon at the 1999 Stockholm Consensus Conference on Setting Global Analytical Goals in Laboratory Medicine [9]. However, since then, a new simpler approach from the EFLM advocates setting APS using the following three models: (1)—clinical outcomes, (2)—BV, and (3)—state-of-the-art performance [9]. We examined both Model 2, as it is widely considered the best general approach [15], and Model 1, in order to assess the APS required to detect a significant or highly significant change in 25(OH)D concentration over one week and three months.

Herein we showed, for the first time, two very important points. The first is that the traditional approach to the generation and application of data on BV is not applicable to 25(OH)D. Indeed, the results of our study show that there is no steady state in 25(OH)D concentrations over time, and that the application of any model based on random variation around homeostatic setting points is inappropriate. This finding may seem obvious, as it has been known for decades that 25(OH)D concentrations observed in summer are higher than those observed in winter [29], but we have demonstrated this for the first time for a cohort of presumably healthy individuals. The immediate application of this finding is that any APS for 25(OH)D based on BV estimates are simply inappropriate.

As a consequence, another approach to determine APS for 25(OH)D examinations is required. The approach that we propose in this paper is to use MU. However, section 5.5.1.4 of ISO 15189 requires that laboratories must determine the standard MU for each measurement procedure in the examination phase used to report the measured quantity values on patients’ samples and then apply a coverage factor to use in routine practice [30]. MU includes components arising from systematic effects. Sometimes estimated systematic effects are not corrected for the method as they should be following metrological principles, but, instead, associated MU components are incorporated [31]. In patient care settings, patient data are typically based on single measurements, and every individual component contributing to the MU of the measurement cannot be corrected for, but generally applicable estimates obtained correctly are valuable in aiding the interpretation of all results. To date, APS for MU have never been published for 25(OH)D. Hence, the second important point of this current work is that we propose APS for MU based on the physiological variation of 25(OH)D concentrations over time. Our results show that, in a European population, 25(OH)D concentrations increased by 2.8% weekly over Spring, and that after 10 weeks, the mean increase was 31.6%. Interestingly, the concentrations observed in the placebo group (*n* = 48) in a randomized controlled trial set in Liège (50.57° N, 5.57° E) during Autumn decreased by a mean of 39.6%, which is approximately the same order of magnitude as the increase observed during Spring in this study [32]. Thus, we believe that the probability of detection of 25(OH)D variation over a certain period of time could become the new paradigm to evaluate 25(OH)D examination methods. Accordingly, we propose that higher order reference methods should present a MU < 1.2%, —and “routine” assays should present a MU < 13.6% to detect a difference (increase) at *p* < 0.05 and 9.6% to detect a difference at *p* < 0.01, this latter APS is, interestingly, almost the same as the current APS for the CV_A_ in the VDSP of 10%. Using a similar strategy, MU targets for higher—order non-reference methods could be based on the probability of detecting a significant change over a five-week period, which, according to the equations and data above, would be a MU < 6.2% at *p* < 0.05. This is totally compatible with the proposal of Stepman and Thienpont [33] in a letter on the first paper published on the MU of 25(OH)D [34].

EQAS are insufficient to assess the entire MU [35,36]. Accuracy-based EQAS does provide higher-order (reference) target concentrations for its materials, which can be used to assess bias/systematic error. This remains an indication, at best, of the true bias of the method being evaluated [35]. From a very practical point of view, the MU could be provided by EQAS providers and by the CDC VDSCP in the certification of the methods. The MU of the VDSCP certified LCMS/MS of CHU de Liège calculated on the 25(OH)D concentrations of the 40 samples received was found to be 5.9%, which is very close to the APS of <6.2% described above. On the other hand, ProBioQual (Lyon, France), a French EQAS provider, proposes the calculation of a MU based on the target value of the peer group, as well as targets based on BV (when available). In 2019, the MU of our routine method (DiaSorin Liaison, Saluggia, Italy) was 16.3%, a little lower than the median of DiaSorin users, but higher than the APS we proposed for a routine method (MU < 13.6%). Finally, the Advisory Panel of the Vitamin D External Quality Assessment Scheme (DEQAS, London, UK) recently proposed providing an MU on the target values assigned by the RMP method on each sample sent to participating laboratories [37]. The combined mean uncertainty of the last distribution in April 2020 (samples 571–575) was 1.3%, which is totally compatible with the MU APS set for the RMP that we proposed.

Standardization programs typically start with an assessment of the examination performance using specimens obtained from the general population, and then include specimens from special subpopulations as recommended by professional organizations. For 25(OH)D, different studies have shown 25(OH)D to behave differently according to the health status of the patient [24,38,39,40]. In situations that do not allow for the assessment of systematic and random effects of health status on examination performance, the use of MU could allow for estimating the importance of those variations according to the APS for MU based on the physiological variation of 25(OH)D. Further studies are needed to assess whether MU will help to better identify and improve the sample-specific bias currently observed with vitamin D assays.

## 5. Limitations

It needs to be noted that the APS suggested here are derived from a European population, and further studies are needed to assess whether these findings can be generalized to populations that are highly diverse with regards to race/ethnicity. We also chose to run an immuno-assay and not our VDSCP certified Liquid Chromatography coupled to tandem Mass Spectrometry method (LCMS-MS) to examine the samples based on several cogent reasons, including the sample volume availability, ability to do long runs, and low imprecision. However, we also examined many of the samples using the LCMS-MS method and the excellent correlation is shown in Supplementary Figure S1. These demonstrate that the impact of the method used to generate the data herein is likely negligible.

## 6. Conclusions

In conclusion, this unique study, which has resulted in a position statement from the IFCC Committee on Bone Metabolism (C-BM), provides evidence that the APS recommended by the clinical laboratory communities and developed by Stöckl et al. more than a decade ago [7] could evolve to include MU. Further studies are needed to assess whether such changes will help to improve the measurement accuracy and reliability of vitamin D assays.

## Figures and Tables

**Table 1:** Examination coefficient of variation (CV_A_), within-subject (CV_I_), and between-subject (CV_G_) biological variation estimates with 95% confidence intervals (CI) of twenty-five-hydroxy vitamin D (25(OH)D).

Dataset	Subgroups	*n* Subject	*n* Results	Sample/ Subject	Replicates *	Mean Value, ng/mL (95% CI)	CV_A_ (%)	CV_I_ (%)	CV_G_ (%)	*n* Results Eliminated %
	Original data	90	1696	9.52	1.96	19.0 (18.6–19.4)	1.7 (1.6–1.8)	17.8		1 subject for Vitamin D consumption from Padua, Italy
All results from 10 collections	All data	46	813	8.93	1.96	22.5 (21.9–23.0)	1.5 (1.4–1.6)	6.3 (5.9–6.8)		875 (51.8%)
	Males	14	242	8.71	1.97	20.5 (19.7–20.8)		6.2 (5.4–7.2)	18.6 (14.1–32.2)	
	Females	32	571	9.03	1.95	23.4 (22.7–24.2)		6.4 (5.8–7.0)	36.2 (28.6–49.2)	
First set of 5 collections (1st–5th)	All data	79	744	4.75	1.97	18.1 (17.5–18.7)	1.9 (1.8–2.1)	6.6 (6.1–7.2)		114 (13.3%)
	Males	33	309	4.70	1.99	15.6 (15.0–16.3)		5.8 (5.1–6.7)	37.1 (28.9–49.5)	
	Females	46	435	4.78	1.96	19.9 (19.0–20.8)		7.1 (6.5–8.0)	51.1 (41.3–66.3)	
Second set of 5 collections (6th–10th)	All data	81	729	4.53	1.97	21.5 (20.9–22.1)	1.5 (1.4–1.7)	6.2 (5.7–6.7)		109 (13.0%)
	Males	33	289	4.39	1.99	19.4 (18.8–20.0)		6.7 (5.9–7.8)	25.0 (20.1–35.1)	
	Females	48	440	4.63	1.96	22.8 (22.0–23.7)		5.8 (5.2–6.4)	39.2 (33.4–52.1)	

* Corresponds to the number of replicates: If we have 10 samples for a subject, with 2 replicates, this means we have 20 numbers. If we have 19 numbers, it means one replicate is not included and the mean number of replicates in this case is 19/10 = 1.9.

## Data Availability

Data presented in this study are available on request from the corresponding author. The data are not publicly available due to intellectual property.

## Supplementary Materials

The following are available online at https://www.mdpi.com/2072-6643/13/2/431/s1, Figure S1: Passing-Bablok regression of 25(OH)D values (ng/mL) obtained with the Fujirebio Lumipulse and the VDSCP-certified LC-MS/MS of the CHU de Liège.
