# Supplementary material for: Analytical Performance Specifications for 25-Hydroxyvitamin D Examinations
Source: Nutrients. 2021 Jan 28;13(2):431. doi: 10.3390/nu13020431 (PMC7911223; doi:10.3390/nu13020431)
Supplement: Supplementary file 1 [file nutrients-13-00431-s001.pdf]

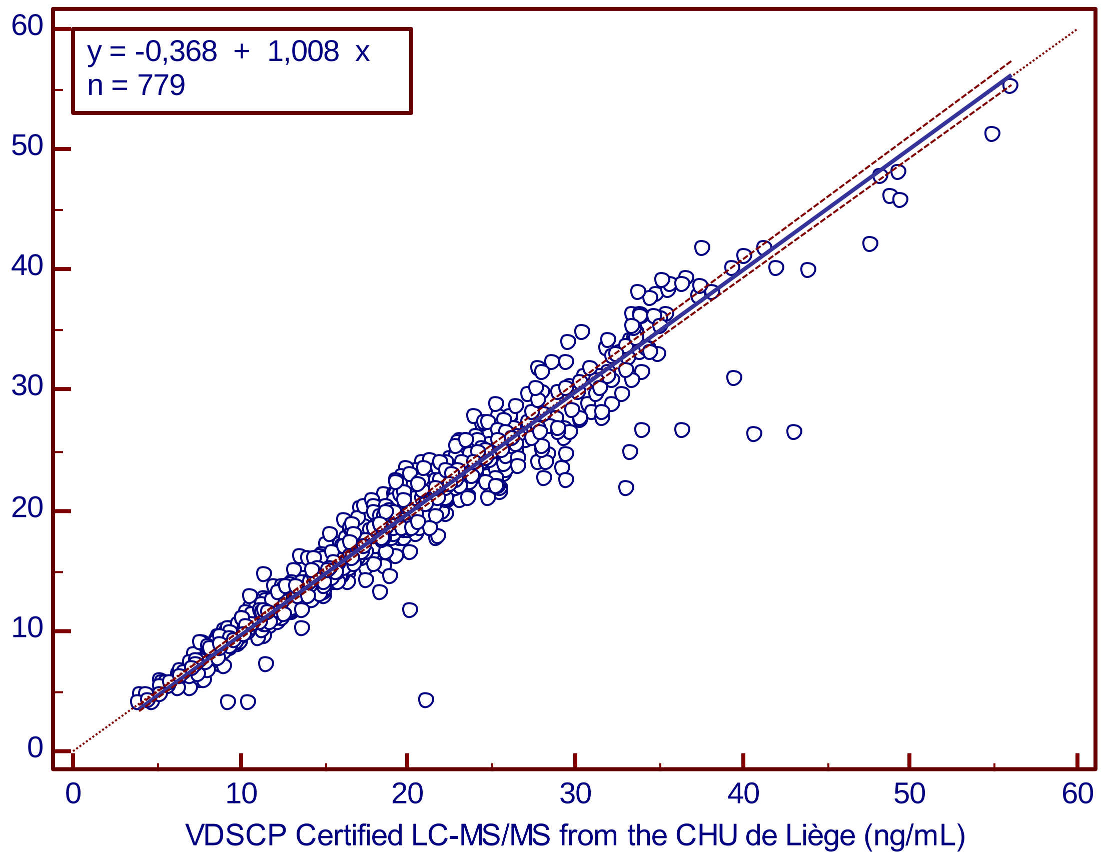

Supplementary Figure S1. Passing-Bablok regression of 25(OH)D values (ng/mL) obtained with the Fujirebio Lumipulse and the VDSCP-certified LC-MS/MS of the CHU de Liège. 25(OH)D: twenty-five-hydroxy vitamin D; VDSCP: Vitamin D Standardization-Certification Program; LC-MS/MS: liquid chromatography tandem mass spectrometry.
